# Simple mathematical model for predicting COVID-19 outbreaks in Japan based on epidemic waves with a cyclical trend

**DOI:** 10.1186/s12879-024-09354-5

**Published:** 2024-05-09

**Authors:** Hiroki Manabe, Toshie Manabe, Yuki Honda, Yoshihiro Kawade, Dan Kambayashi, Yoshiki Manabe, Koichiro Kudo

**Affiliations:** 1 Shitennoji University, 3-2-1 Gakuenmae, Habikino City, 583-8501 Osaka Japan; 2 Nagoya City University School of Data Science, Nagoya City, Aichi Japan; 3 Nagoya City University Graduate School of Medicine, Nagoya City, Aichi Japan; 4 Showa Pharmaceutical University, Machida, Tokyo Japan; 5 Tokyo University Graduate School of Engineering, Tokyo, Japan; 6 Waseda University Organization Regional and inter-regional Studies, Tokyo, Japan; 7 Kawakita General Hospital, Tokyo, Japan

**Keywords:** COVID-19, SARS-CoV-2, Prediction model, Mathematical model, Machine learning technique, Periodicity, Rising trend line, Epidemic wave, Autocorrelation coefficient, Correlogram

## Abstract

**Background:**

Several models have been used to predict outbreaks during the COVID-19 pandemic, with limited success. We developed a simple mathematical model to accurately predict future epidemic waves.

**Methods:**

We used data from the Ministry of Health, Labour and Welfare of Japan for newly confirmed COVID-19 cases. COVID-19 case data were summarized as weekly data, and epidemic waves were visualized and identified. The periodicity of COVID-19 in each prefecture of Japan was confirmed using time-series analysis and the autocorrelation coefficient, which was used to investigate the longer-term pattern of COVID-19 cases. Outcomes using the autocorrelation coefficient were visualized via a correlogram to capture the periodicity of the data. An algorithm for a simple prediction model of the seventh COVID-19 wave in Japan comprised three steps. Step 1: machine learning techniques were used to depict the regression lines for each epidemic wave, denoting the “rising trend line”; Step 2: an exponential function with good fit was identified from data of rising straight lines up to the sixth wave, and the timing of the rise of the seventh wave and speed of its spread were calculated; Step 3: a logistic function was created using the values calculated in Step 2 as coefficients to predict the seventh wave. The accuracy of the model in predicting the seventh wave was confirmed using data up to the sixth wave.

**Results:**

Up to March 31, 2023, the correlation coefficient value was approximately 0.5, indicating significant periodicity. The spread of COVID-19 in Japan was repeated in a cycle of approximately 140 days. Although there was a slight lag in the starting and peak times in our predicted seventh wave compared with the actual epidemic, our developed prediction model had a fairly high degree of accuracy.

**Conclusion:**

Our newly developed prediction model based on the rising trend line could predict COVID-19 outbreaks up to a few months in advance with high accuracy. The findings of the present study warrant further investigation regarding application to emerging infectious diseases other than COVID-19 in which the epidemic wave has high periodicity.

**Supplementary Information:**

The online version contains supplementary material available at 10.1186/s12879-024-09354-5.

## Background

Following reports of the first patients with viral pneumonia caused by COVID-19 in December 2019 from Wuhan City in Hubei Province, China, the disease rapidly spread throughout the world, leading to the COVID-19 pandemic [1, 2]. In Japan, the first patient with COVID-19 was reported on January 15, 2020. This was the second imported case from China, following those from Thailand [3, 4]. Since then, people in Japan have experienced eight epidemic waves of COVID-19, with approximately 33.5 million laboratory-confirmed cases and 74,000 deaths as of March 31, 2023 [5]. Each time an epidemic wave occurs, the number of laboratory-confirmed COVID-19 cases increases rapidly. This rapid elevation in the number of cases caused increased fears of health system collapse, with hospitals experiencing difficulty treating patients with diseases other than COVID-19, such as myocardial infarction and heart failure [6], as well as an increase in the number of patients with out-of-hospital cardiac arrest [7]. However, if the timing and shape of an epidemic wave could be predicted in advance with high accuracy, hospitals and health care systems could better prepare according to the predicted number of cases and expected time frame.

Several prediction models have been proposed for infectious diseases, including the historical mathematical model based on differential equations such as the SIR (susceptible, infectious, recovered) model [8]; models involving time-series analysis with use of past data to predict the future, such as the autoregressive integrated moving average (ARIMA) and seasonal ARIMA (SARIMA) models [9–11]; and models that learn data patterns for prediction and classification, such as the Prophet model [12, 13]. Additionally, the effective application of outbreak prediction or forecasting models is crucial for obtaining insightful information regarding the transmission dynamics of a disease and its consequences. However, standard prediction models that deliver accurate results have not yet been established [14]. Various factors increase the uncertainty of prediction models, including known and unknown variables, differences in population/behavioral complexity in different geopolitical areas, people’s vaccination status, the evolution of new strains, medical measurements, and variations in containment strategies [15–17]. Therefore, developing a standard prediction model for COVID-19 outbreaks that matches the real-world data with adjustment for these risk factors is challenging. Under these conditions, a highly accurate prediction model for COVID-19 in Japan is still lacking. In a comparison among the 47 prefectures of Japan, the speed of increase in the number of cases differs according to prefecture [5]. A prediction model of COVID-19 for Japan must be adjusted to the conditions in each prefecture; therefore, a highly accurate, tailor-made prediction model must incorporate the local conditions. However, it can be hypothesized that if medical organizations and local governments in each location can produce accurate epidemic predictions, this would contribute to the preparation of countermeasures against COVID-19 throughout the health system that match the conditions in each region.

The aim of the present study was to predict COVID-19 outbreaks in Japan using a simple mathematical model.

## Methods

### Dataset

We used data that were made openly available by the Ministry of Health, Labour and Welfare of Japan from the first confirmed COVID-19 case on January 16, 2020 up to May 8, 2023 [18]. During this period in Japan, successive variations in the progression and containment of the epidemic, or waves, were observed eight times. Data for daily counts of new COVID-19 cases at both national and prefectural levels were collected and used for developing the model.

The end date of each epidemic wave was defined as the week with the minimum number of weekly cases after the peak of the epidemic (in which the number of weekly cases was greater than that in the following weeks). Using this definition, the duration of each epidemic wave in Japan was as follows. Wave 1 was from January 16, 2020 to May 24, 2020; Wave 2: May 25, 2020 to September 27, 2020; Wave 3: September 28, 2020 to February 28, 2021; Wave 4: February, March 1, 2021 to June 20, 2021; Wave 5: June 21, 2021 to October 3, 2021; Wave 6: November 29, 2021 to June 19, 2022; Wave 7: June 20, 2022 to October 9, 2022; and Wave 8 was from October 10, 2022 to April 2, 2023.

### Assessing the periodicity of epidemic waves using time-series analysis and the autocorrelation coefficient

We used time-series analysis and the autocorrelation coefficient (ACF) to examine the periodicity of COVID-19 epidemic waves in Japan. A time-series analysis necessitates decomposing the data into fundamental elements: trends that capture global trends and irregular, short-term fluctuations (called noise). Given the pronounced short-term periodicity influenced by weekdays among COVID-19 cases in Japan, a moving average method was adopted. This involved calculating a 7-day average centered around each day to smooth out variations, treating it as the daily count of newly confirmed cases.

The ACF was applied to investigate longer-term patterns of COVID-19 cases [19]. The ACF is used to calculate the correlation coefficient between the original data and data shifted by specific time lags (referred to as lags), aiming to ascertain the presence of periodicity. The ACF outcomes were visualized using a correlogram that captures the periodicity of the data, representing the correlation coefficients between the original data x and the time-shifted data y. This is a plot with lags on the horizontal axis and autocorrelation on the vertical axis, illustrating the relationship. The graph initiates from lag 0, where the correlation coefficient is 1 from a comparison with the identical dataset. Generally, as the lag increases, the correlation tends to attenuate, although instances arise where the strength of the correlation re-emerges. This phenomenon signifies autocorrelation, serving as a guide for exploring periodicity. We conducted time-series analysis of changes in the ACFs after January 2020. Then, the collected data were used to derive a predictive framework for epidemic trends.

### Algorithm for a simple prediction model of the seventh COVID-19 wave in Japan

**Fig. 1:**
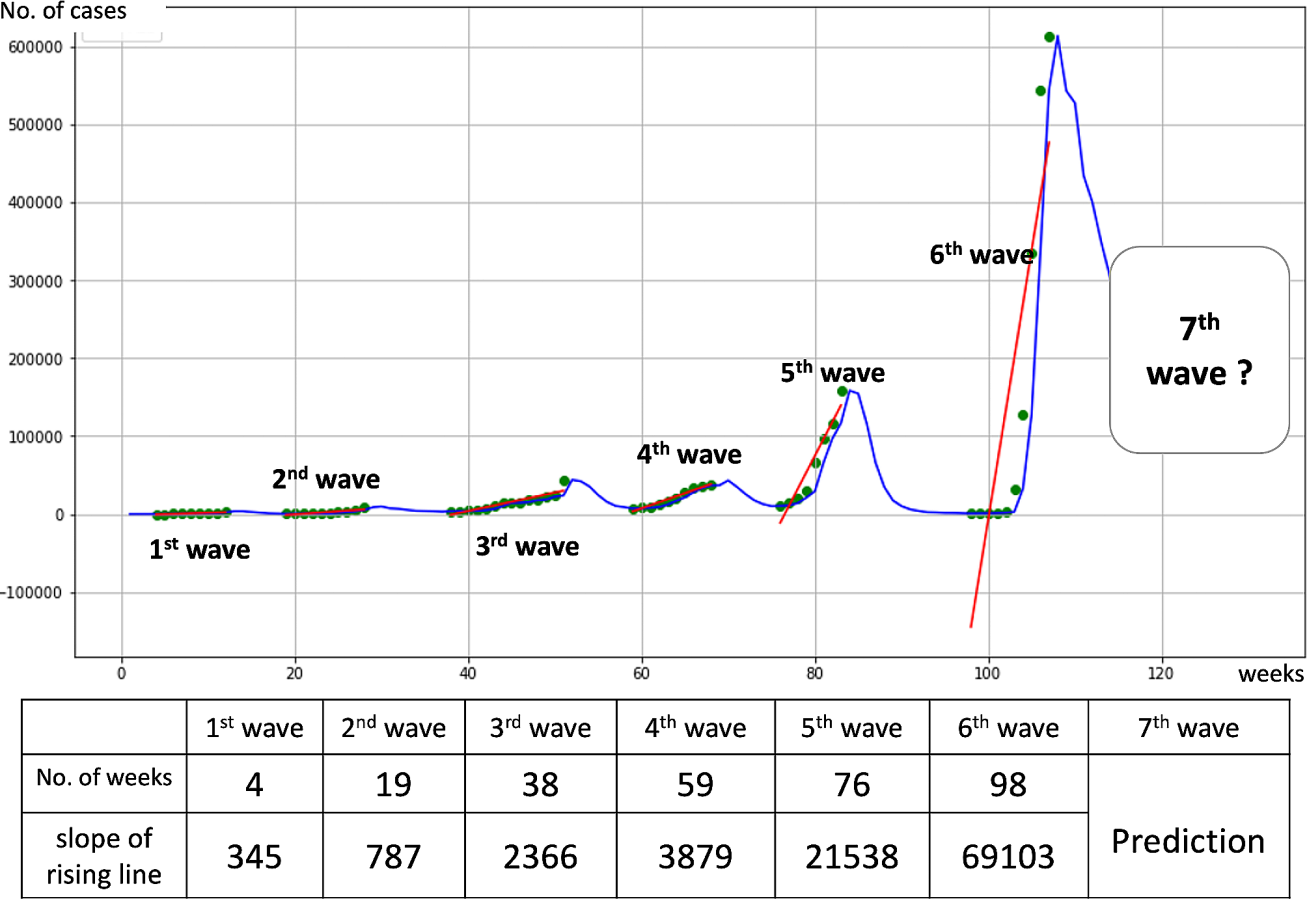
Epidemic waves of COVID-19 in Japan (blue line) with the rising trend line (red line)

**Step 1**. A time series of COVID-19 case datasets (i.e., daily new confirmed cases) from January 15, 2020 to April 23, 2023 was generated in Python programming language (Ver. 3.0.14) using pandas (ver. 1.2.4) to conduct the tabular data analysis. We used a function of a linear regression model from the machine learning library scikit-learn (ver. 0.24.1) to fit the regression lines for each epidemic wave, which we denoted the “rising trend line. (Fig. 1). The data were visualized using matplotlib (ver. 3.3.4) and seaborn (0.11.1).

The next epidemic wave might be predicted on the basis of regular changes in the slope of this line.

**Fig. 2:**
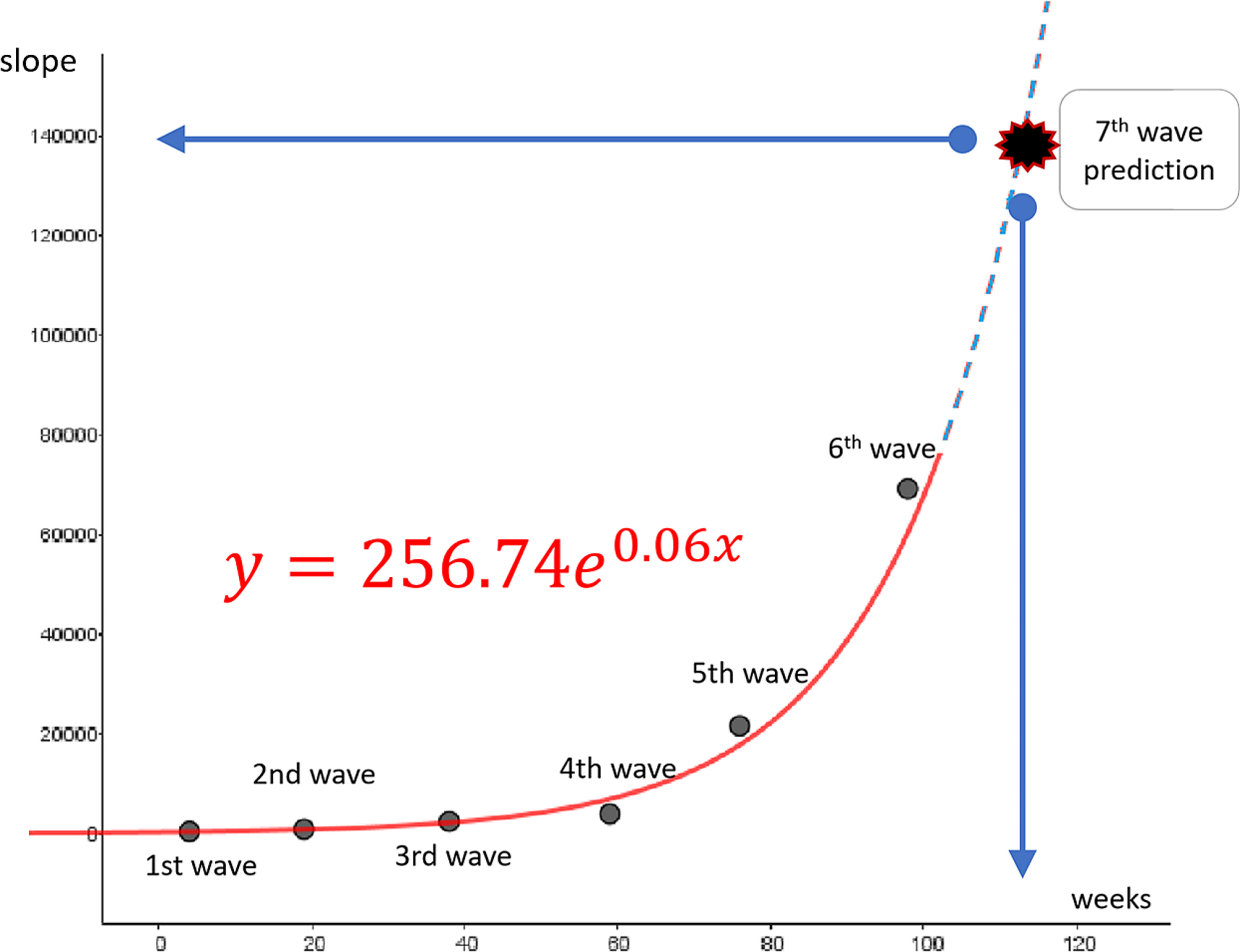
Prediction diagram of speed of increase in number of COVID-19 cases and timing of outbreak

**Step 2**. We then identified an exponential function with good fit from the data for rising straight lines up to the sixth epidemic wave and calculated the timing for the rise of the seventh wave and its speed of spread using the GeoGebra’s function for applying an exponential model of bivariate regression analysis (GeoGebra Classic 5.2.826.0-d, International GeoGebra Institute, Linz, Austria). Regression analysis was performed after plotting the data calculated in step 1 on a coordinate plane to fit the logistic growth curves. The obtained model is shown in Fig. 2.

The surge timing was predicted using the average interval between waves up to the sixth wave, and the exponential function was used to calculate its expansion speed. After the calculated data were plotted on a coordinate plane, regression analysis was performed, and the function with a good fit was adopted as model (1).

1 $$\text{y=256.74}{e}^{0.06\text{x}}$$

The average interval between each epidemic wave was 19 weeks (95% confidence interval, 13.1–25.0). We divided the duration from the time of occurrence of an epidemic to the time of its peak into two periods, a rising period and peak period. We then calculated the slope of the rising trend line. We predicted the seventh wave during the convergence period of the sixth wave. This period was longer than those in the previous epidemic period, so we added 10 weeks to the start time of the seventh wave. The coefficients used to predict the slope of the rising trend line are shown in Additional file 1.

**Step 3**. A logistic growth model is often used to fit the time series analysis in studies of infectious diseases [20–22] (2).

2 $$f\left(t\right)=\frac{1}{1+{e}^{-t}}$$

Using the logistic growth model, the variable t represents the number of days (duration) in each epidemic wave; a_i_ is the number of infected people in each epidemic; b_i_ is the inflection point of the curve representing the transition (number of days at the peak in an epidemic wave); and the speed of infection. Using c_i_ as a coefficient to adjust, the total number of infected people in that epidemic wave is expressed by the following formula.

3 $${f_i}\left( t \right) = \frac{{{a_i}}}{{1 + {e^{\frac{{{b_i} - t}}{{{c_i}}}}}}}$$

The following equation, which differentiates this with respect to the time variable t, can be expressed as the daily number of cases. Then, the variables a_*i*_, b_*i*_, and c_*i*_ in each epidemic wave are calculated as follows:

4 $$\frac{{d{f_{i\left( t \right)}}}}{{dt}} = \frac{{{a_i}{e^{\frac{{{b_i} - t}}{{{c_i}}}}}}}{{{c_i}{{(1 + {e^{\frac{{{b_i} - t}}{{{c_i}}}}})}^2}}}$$

We used Python programming language (Ver. 3.0.14) for the analytical process throughout the present study. For all analyses, significance levels were two-tailed, and *p* < 0.05 was considered statistically significant.

## Results

### Periodicity of COVID-19 in Japan using time-series analysis and the autocorrelation coefficient (ACF)

**Fig. 3:**
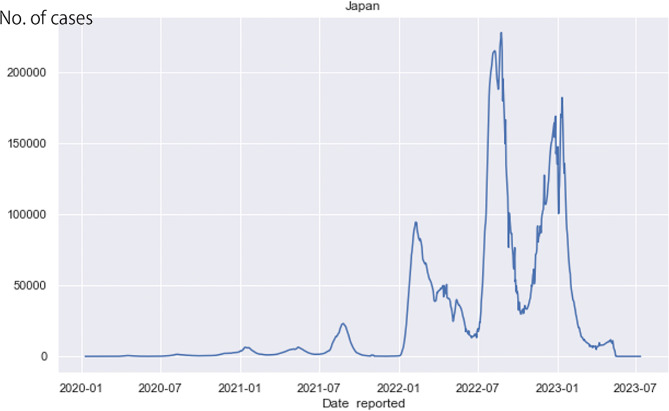
Temporal distribution of COVID-19 cases, January 2020–March 2023

The temporal distribution of COVID-19 cases during the whole observational period (January 2020 to March 2023) is shown in Fig. 3.

We used advanced machine learning techniques to ascertain and visually represent the epidemic waves with a rising trend line (Fig. 1) We also assessed the distribution of COVID-19 cases with a rising trend line in Japan’s 47 prefectures; different trends among epidemic waves in each prefecture can be observed (Additional file 2).

**Fig. 4:**
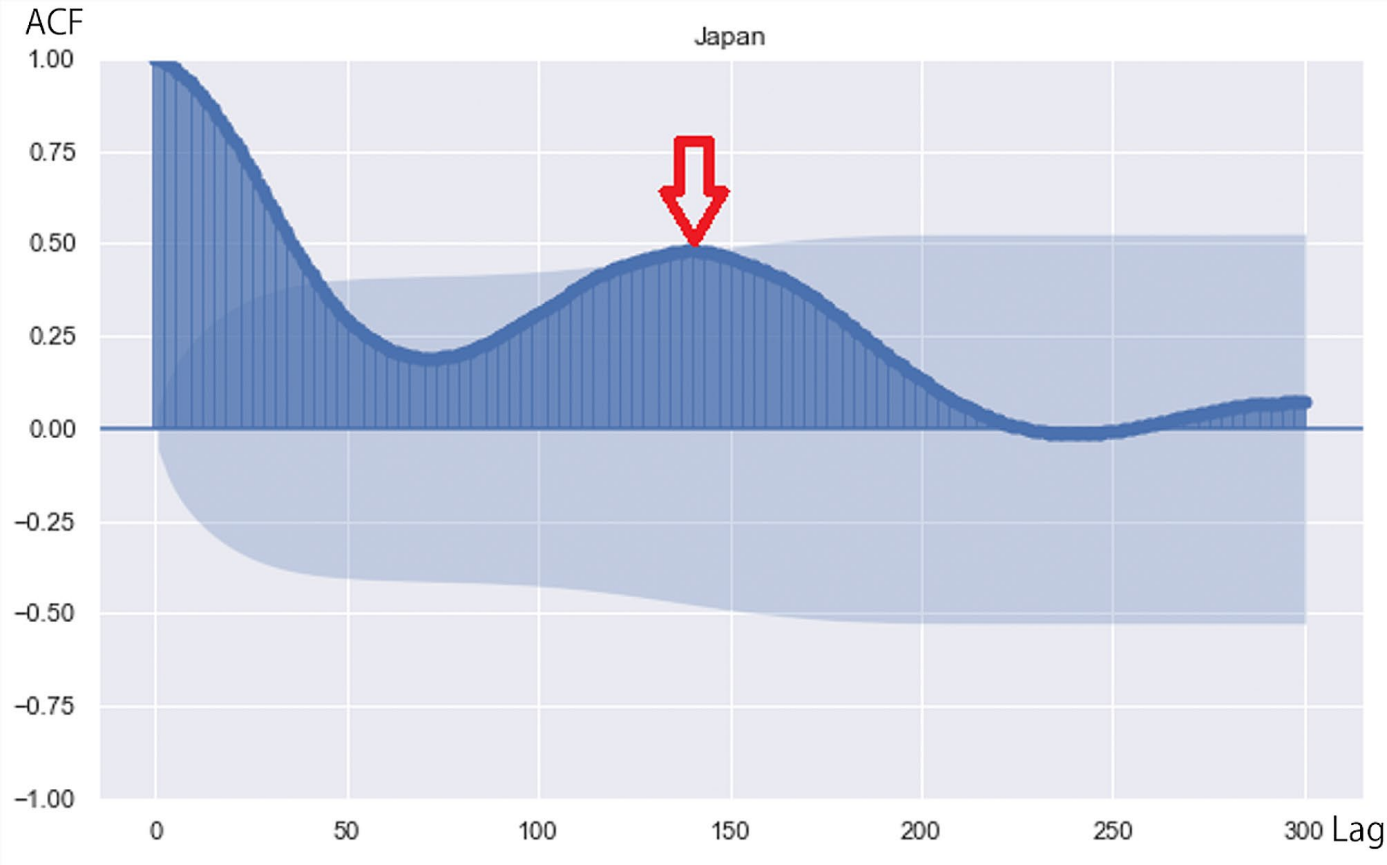
Correlogram using data from January 2020 to March 2023 for COVID-19 cases in Japan. Red arrow indicates the peak of lag. Light blue band indicates 95% confidence interval. Values outside the band (dark blue), which are the autocorrelation coefficient, are statistically significant

Figure 4 shows the correlogram using the whole observational period (up to March 31, 2023).

Between January 16, 2020 and May 8, 2023, the correlation coefficient was approximately 0.5, indicating significant periodicity. The peak of lag was approximately 140 (red arrow). Therefore, the spread of infection was repeated in a cycle of approximately 140 days, with high periodicity.

### Prediction of the seventh COVID-19 wave and its accuracy

**Fig. 5:**
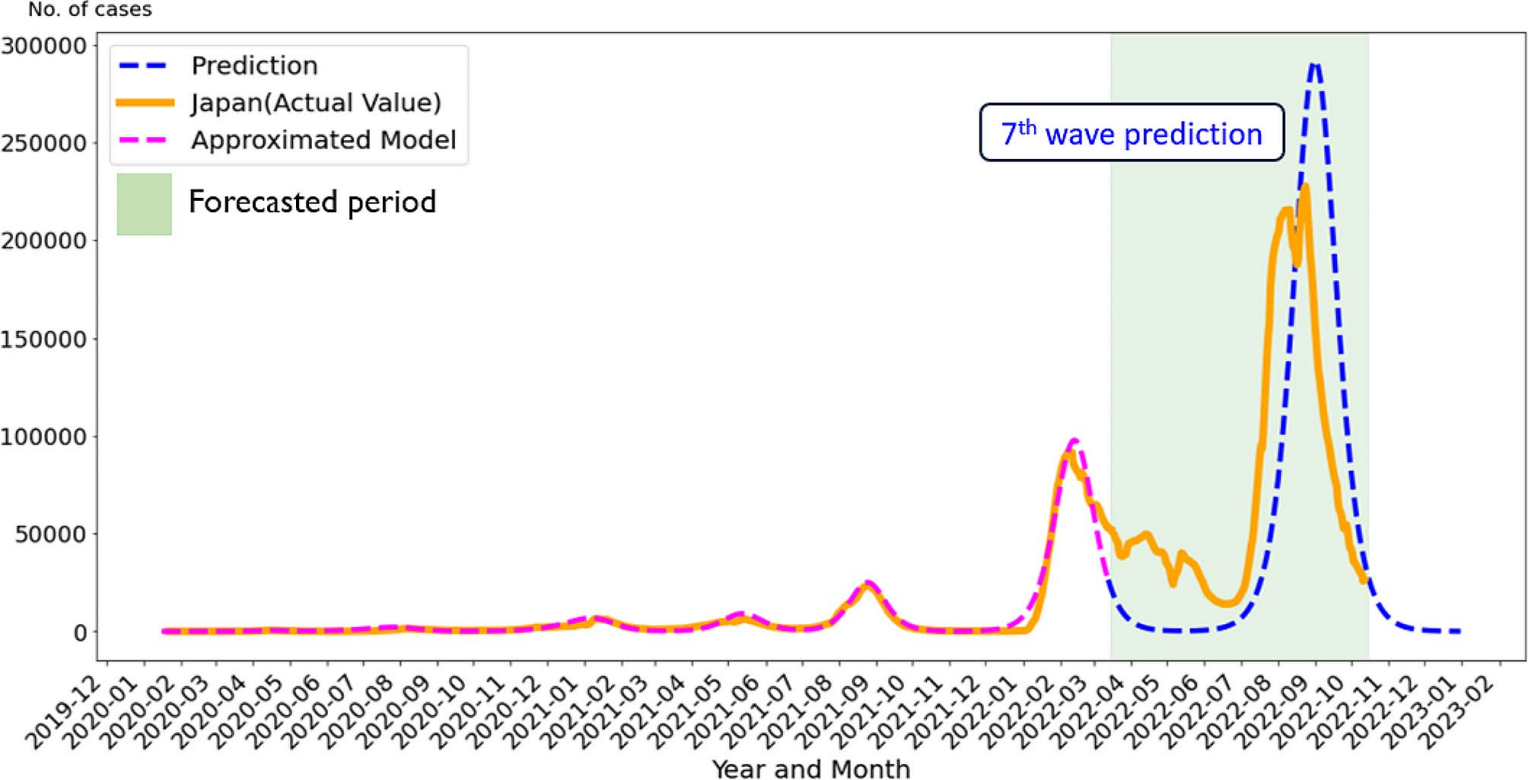
Distribution of COVID-19 cases and simulated wave developed using a prediction model. Orange line: actual distribution of COVID-19 cases. Pink line: distribution using the approximated model. Blue line: simulated wave developed using prediction. Gray shade: Forecasted period

The epidemic curves using actual data for the number of infected cases and the approximate model are shown in Fig. 5, including the time of prediction of the seventh wave (March 15, 2022) and the simulated wave using the prediction model, as well as the actual number of cases after the time of prediction.

The actual distribution of COVID-19 cases and the distribution using the approximated model, which were used as the basic tool for developing the prediction model, presented similar distributions by mid-March 2022 (Fig. 5). In the predicted seventh wave, although the starting time and peak time of the epidemic were slightly behind, the mean absolute percent error (MAPE) during March 13, 2022 to October 12, 2022 indicated 53.5% but 20.0% for the 3 months from July 1, 2022 to October 1, 2022 during the outbreak and 13.9% for the 1-month period from July 1, 2022 to August 1, 2022 during the expansion period. Therefore, although this provides the rationale for setting it to 200 days, if there had been a 15-day difference, it would have been possible to predict the outbreak and spread with a high degree of accuracy.

## Discussion

We constructed a simple prediction model by combining the depicted rising trend line. Our model showed a high degree of accuracy, especially when the distribution of COVID-19 cases had substantial periodicity. The spread of COVID-19 in Japan was repeated in a cycle of approximately 140 days, with high periodicity.

After the emergence of the COVID-19 pandemic, SARS-CoV-2 spread rapidly across countries worldwide, including Japan. It threatened people’s daily lives and caused medical care challenges such as health system collapse, which would make it impossible for patients with COVID-19 to be treated in a hospital. However, if it were possible to predict the timing of a future outbreak with high accuracy, as well as the trends in the number of cases over time, targeted infection control measures could be efficiently planned and the medical system could adequately prepare. We constructed a model to predict the surge timing of an epidemic wave using an exponential function, which yielded empirical evidence to support this model up to the seventh COVID-19 wave in Japan. The periodicity in SARS-CoV-2 transmission may stem primarily from changes in the implemented public health and social measures, adherence levels, antigenic drift, and seasonality influenced by environmental factors. However, because COVID-19 is an emerging infectious disease, risk factors that affect the spread of infection have not been clearly elucidated. Additionally, COVID-19 infection may be transmitted from asymptomatic or pre-symptomatic individuals [23] and many cases of reinfection have been reported [24]. Owing to these issues, it is considered difficult to predict COVID-19 outbreaks with high accuracy and in a timely fashion using conventional SIR models and models using the basic reproduction number or effective reproduction number.

From our previous studies, we have seen that some emerging and re-emerging infectious diseases have a high cyclic trend [25, 26]. We hypothesized that if periodicity exists for COVID-19 cases in Japan, we can predict the timing of a future outbreak. Although the periodicity varied slightly among prefectures, the spread of COVID-19 across Japan exhibited a cycle of approximately 140 days, with high periodicity. This number is reasonable within the context of the COVID-19 pandemic in Japan, where epidemic waves occurred mainly during the summer and winter. We then calculated and plotted the rising trend line for the next epidemic wave, after the peak of the previous epidemic wave had ended. The timing from the calculation differed from that of a graphical model using a line rising with the increase in the number of cases per day [27]. Parag et al. derived a novel method that can estimate the probability for the end of an epidemic [28]. Incorporating this method into our prediction model confers the possibility of analyzing the timing of convergence of the epidemic wave after the peak and potentially analyzing the timing of convergence of post-peak epidemic waves, leading to enhanced analysis by the prediction model. Thus, we would be able to predict the number of COVID-19 cases over time during the next epidemic wave months in advance. These models are simple; however, by combining the calculation methods for the cyclic trend and the rising trend line. This method could predict the starting time, peak, and number of cases in the seventh COVID-19 wave in Japan, during the previous wave. However, more work is needed to verify that our findings apply to other locations and other time frames.

We confirmed that the shape and rising trend line of the epidemic wave differed depending on each prefecture in Step 2 of the algorithm. However, in Japan, the same COVID-19 countermeasures were taken nationwide under government initiatives. Therefore, in Step 3 of the algorithm, we used national data and analyzed data throughout Japan to build an infection prediction model, in preparation for building different models for each prefecture and region in the future. We also found that each prefecture in Japan had a different periodicity. However, periodicity was analyzed by integrating national data, and analysis that considered periodicity in each prefecture was not carried out. When examining periodicity using a correlogram, the periodicity may change depending on the time of the analysis owing to changes in the amount of data used; it was not possible to reliably confirm the periodicity of 140 days using national data for Japan. Additionally, during a prolonged epidemic period, it is unclear whether it can be assumed that the speed of increase will consistently follow the proposed exponentially increasing pattern in the future, especially after society transitions to the normal endemic phase of SARS-CoV-2 circulation. Although there are various known and unknown variables and factors related to the conditions leading to infection, we developed our prediction model using solely case incidence data. It is unclear which of these factors are true influencing factors, and their weight. Additionally, mutations of the virus, the availability of vaccines and people’s vaccination status, and individual knowledge about prevention measures are related to the spread of COVID-19. However, it is difficult to obtain such data in a timely manner, because these data change constantly over time. Moreover, there are limits to available data related to the characteristics of COVID-19, such as who an infected person infects, how many people they infect, and the number of pre-symptomatic and asymptomatic cases for each variant. In the future, we are considering using available data from medical institutions and public health centers, calculating predicted values for unavailable data, and developing a predictive model using these data.

Despite these limitations, it is crucial to consider the various patterns of prediction models in preparation for future emerging infectious diseases to protect the health of populations worldwide.

## Conclusions

Our simple model, which uses periodicity and the rising trend line, showed that if past outbreaks have periodicity, the spread of COVID-19 can be predicted up to a few months in advance. The study findings suggest the possibility of predicting the starting point of a future infectious disease outbreak and the number of infected individuals, contributing to early policy decision-making and advanced health system preparation. The results suggest that our developed simple mathematical prediction model can facilitate tailor-made epidemic prediction of COVID-19 outbreaks in the future.

### Electronic supplementary material

Below is the link to the electronic supplementary material.

Supplementary Material 1

Supplementary Material 2

## Data Availability

Data is provided within the manuscript or supplementary information files.

## Abbreviations

COVID-19: Coronavirus disease 2019
SARS-CoV-2: Severe acute respiratory syndrome coronavirus 2
WHO: World Health Organization
ACF: Autocorrelation coefficient
