# Supplementary material for: Simple mathematical model for predicting COVID-19 outbreaks in Japan based on epidemic waves with a cyclical trend
Source: BMC Infect Dis. 2024 May 9;24:465. doi: 10.1186/s12879-024-09354-5 (PMC11080248; doi:10.1186/s12879-024-09354-5)

Additional file 2.

S1. Fig. The distribution of COVID-19 cases in Japan's 47 prefectures with the rising trend line

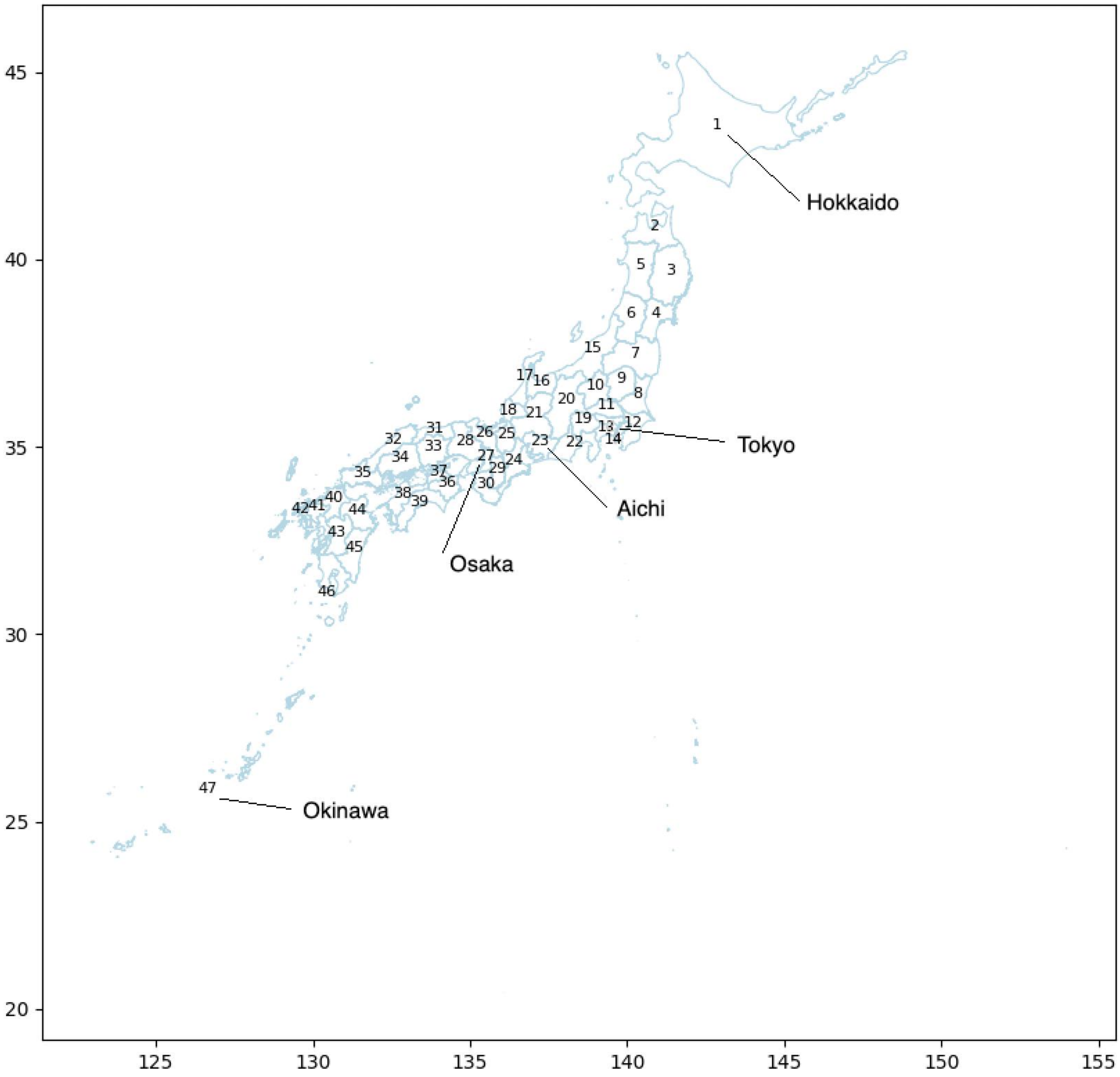

--- 0. Over the nation ---
1st [348.3] ,2nd [787.9] ,3th [2366.7] ,4th [3874.7] ,5th [21540.7] ,6th [67810.8]

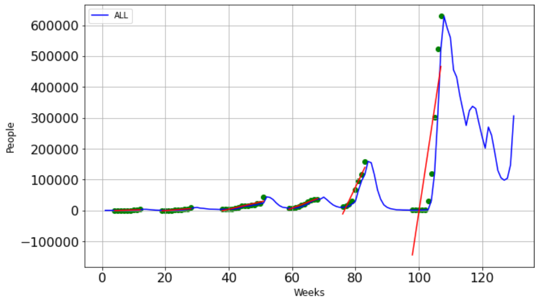

--- 1. Hokkaido ---
1st [14.5] ,2nd [7.8] ,3th [149.4] ,4th [651.2] ,5th [487.1] ,6th [2713.4]

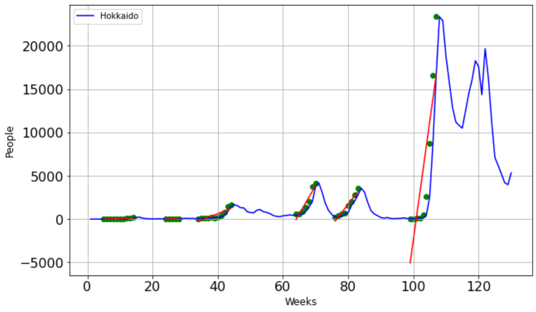

--- 2. Aomori ---

1st [3.] ,2nd [-2.] ,3th [67.] ,4th [14.7] ,5th [134.1] ,6th [345.3]

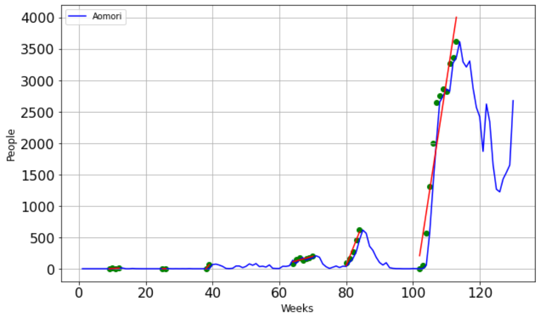

--- 3. Iwate ---

1st [0.] ,2nd [0.9] ,3th [11.] ,4th [53.] ,5th [26.2] ,6th [155.8]

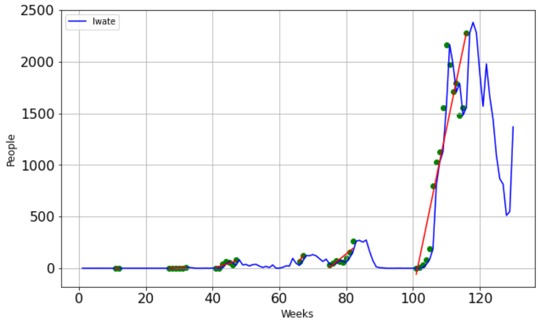

--- 4. Miyagi ---

1st [12.5] ,2nd [5.3] ,3th [36.6] ,4th [174.5] ,5th [165.2] ,6th [773.2]

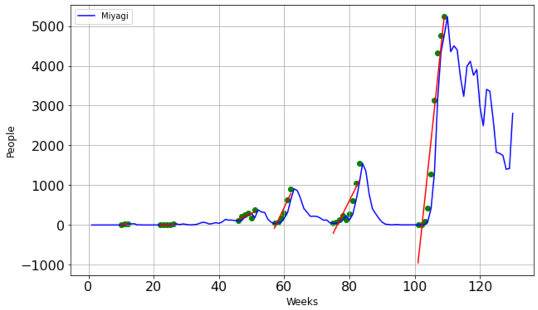

--- 5. Akita ---

1st [1.] ,2nd [17.] ,3th [6.1] ,4th [16.7] ,5th [56.9] ,6th [124.9]

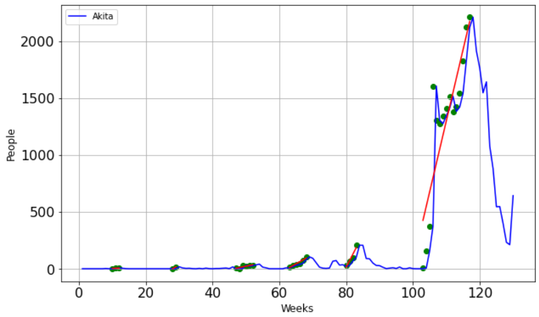

--- 6. Yamagata ---

1st [14.] ,2nd [0.] ,3th [14.2] ,4th [104.] ,5th [75.7] ,6th [372.9]

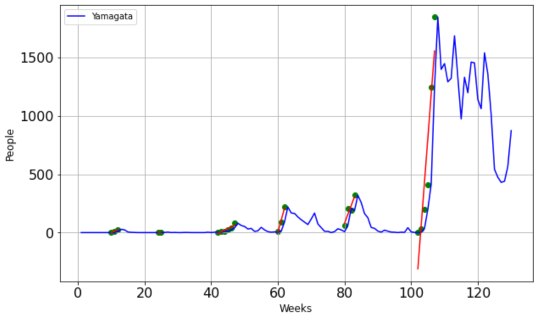

--- 7. Fukushima ---

1st [8.] ,2nd [5.9] ,3th [19.] ,4th [32.6] ,5th [215.6] ,6th [215.2]

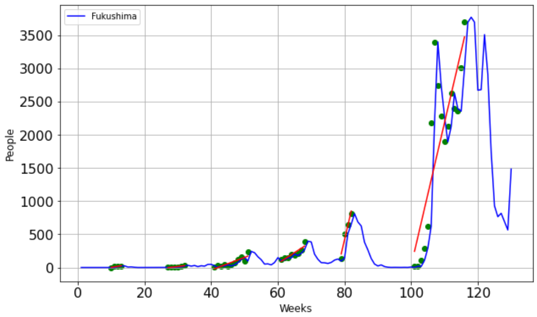

--- 8. Ibaraki ---

1st [16.1] ,2nd [9.9] ,3th [30.6] ,4th [26.1] ,5th [367.5] ,6th [1141.4]

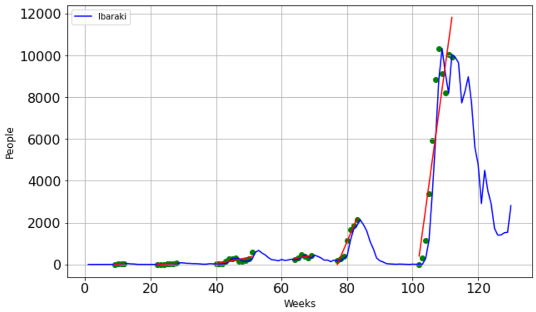

--- 9. Tochigi ---

1st [2.8] ,2nd [7.5] ,3th [60.8] ,4th [28.3] ,5th [253.6] ,6th [779.5]

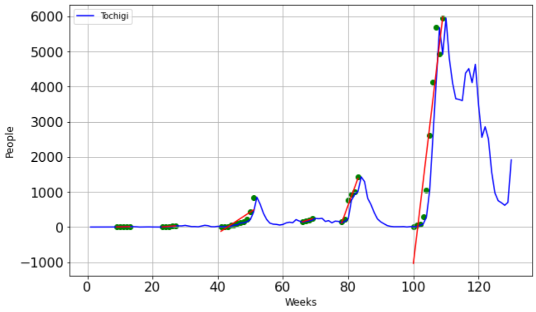

--- 10. Gunma ---
1st [10.] ,2nd [5.8] ,3th [40.3] ,4th [50.5] ,5th [226.4] ,6th [512.3]

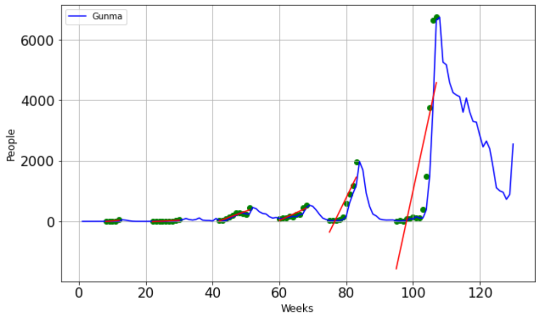

--- 11. Saitama ---
1st [39.2] ,2nd [63.4] ,3th [138.] ,4th [131.8] ,5th [1757.2] ,6th [4408.2]

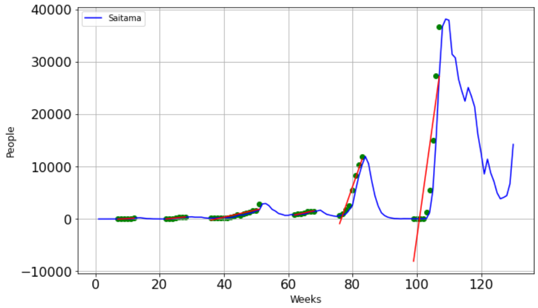

--- 12. Chiba ---
1st [40.1] ,2nd [47.1] ,3th [191.6] ,4th [51.7] ,5th [932.] ,6th [2692.1]

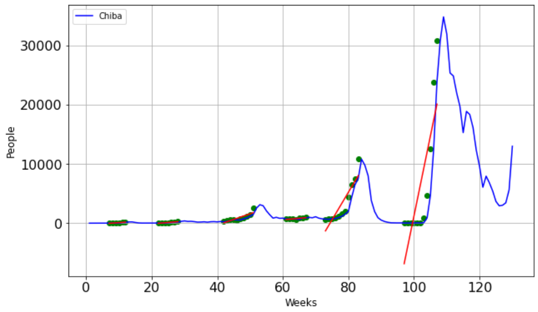

--- 13. Tokyo ---
1st [134.8] ,2nd [246.6] ,3th [805.1] ,4th [539.2] ,5th [3963.8] ,6th [15737.8]

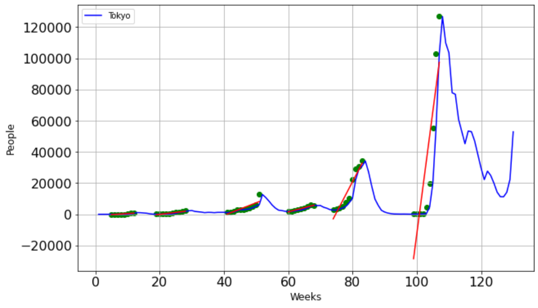

--- 14. Kanagawa ---
1st [31.2] ,2nd [85.4] ,3th [469.3] ,4th [183.] ,5th [1824.1] ,6th [5511.]

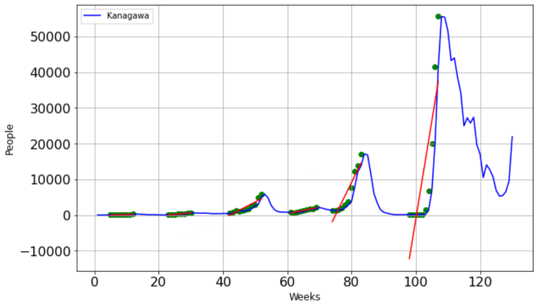

--- 15. Niigata ---
1st [0.9] ,2nd [4.4] ,3th [6.6] ,4th [20.1] ,5th [104.] ,6th [244.]

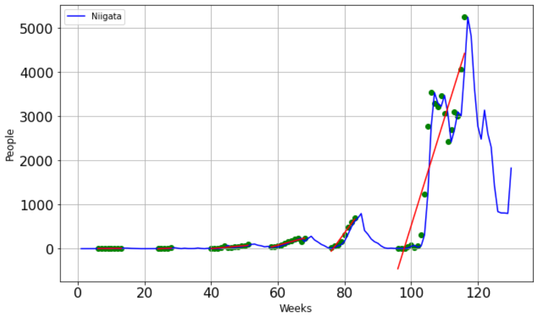

--- 16. Toyama ---
1st [19.6] ,2nd [5.] ,3th [9.1] ,4th [15.5] ,5th [113.9] ,6th [364.2]

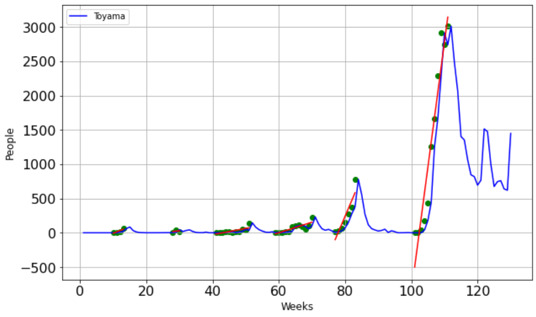

--- 17. Ishikawa ---
1st [34.5] ,2nd [20.9] ,3th [9.9] ,4th [41.2] ,5th [104.] ,6th [402.1]

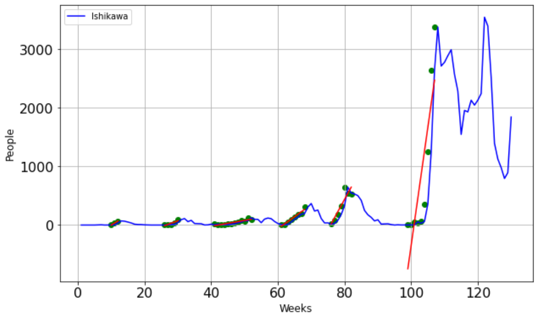

--- 18. Fukui ---
1st [28.] ,2nd [51.] ,3th [2.6] ,4th [13.9] ,5th [20.1] ,6th [269.3]

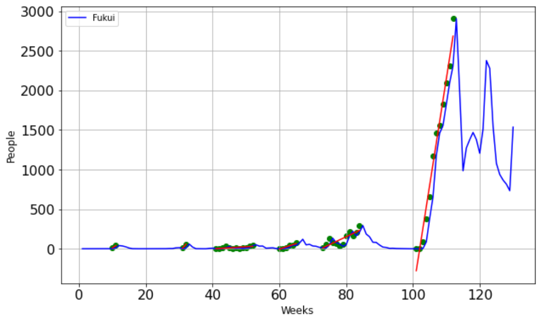

--- 19. Yamanashi ---
1st [9.] ,2nd [5.4] ,3th [13.6] ,4th [7.6] ,5th [125.8] ,6th [223.9]

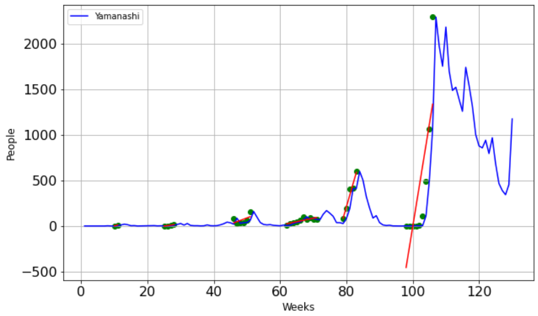

--- 20. Nagano ---
1st [6.5] ,2nd [6.5] ,3th [17.9] ,4th [53.8] ,5th [138.3] ,6th [314.5]

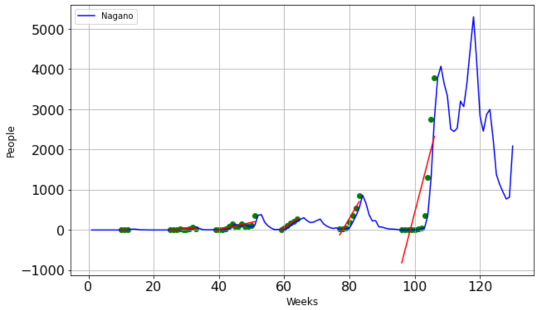

--- 21. Gifu ---
1st [17.8] ,2nd [29.] ,3th [45.4] ,4th [99.6] ,5th [277.2] ,6th [1073.1]

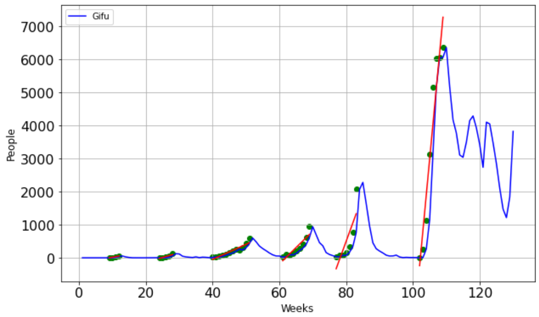

--- 22. Shizuoka ---
1st [12.] ,2nd [22.2] ,3th [29.6] ,4th [44.5] ,5th [423.] ,6th [2424.]

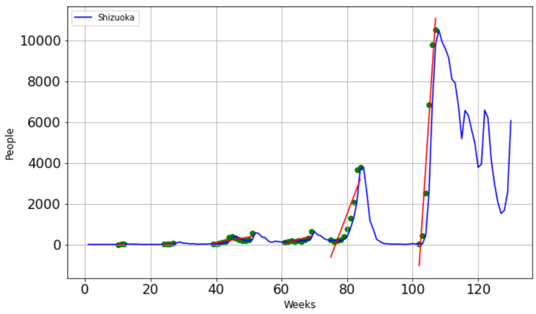

--- 23. Aichi ---
1st [5.7] ,2nd [259.6] ,3th [149.7] ,4th [376.2] ,5th [1552.3] ,6th [8229.4]

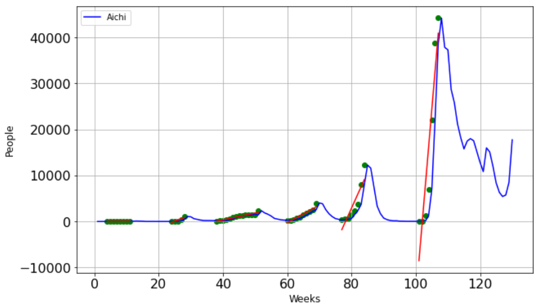

--- 24. Mie ---
1st [5.7] ,2nd [15.6] ,3th [16.1] ,4th [37.8] ,5th [284.7] ,6th [841.6]

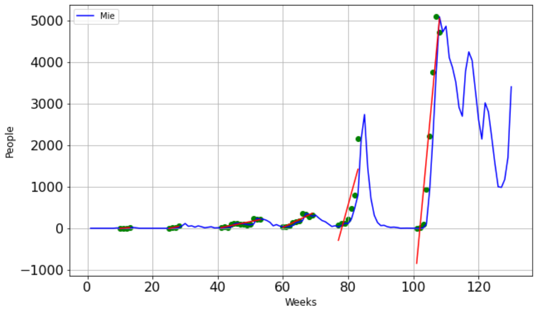

--- 25. Shiga ---
1st [8.] ,2nd [23.5] ,3th [15.3] ,4th [52.7] ,5th [217.] ,6th [732.2]

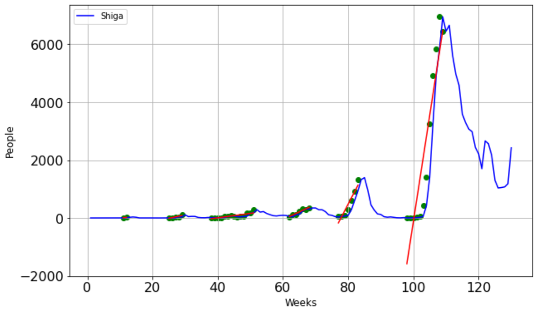

--- 26. Kyoto ---
1st [35.5] ,2nd [23.1] ,3th [71.] ,4th [123.5] ,5th [478.2] ,6th [2211.4]

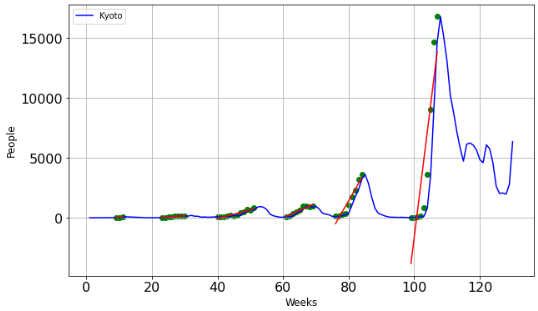

--- 27. Osaka ---
1st [54.4] ,2nd [163.5] ,3th [198.8] ,4th [1219.5] ,5th [1905.6] ,6th [13072.9]

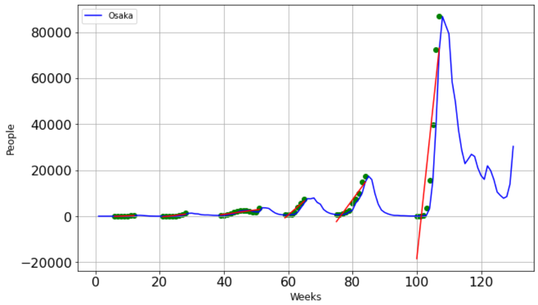

--- 28. Hyogo ---
1st [23.] ,2nd [47.5] ,3th [125.8] ,4th [526.9] ,5th [884.6] ,6th [3211.2]

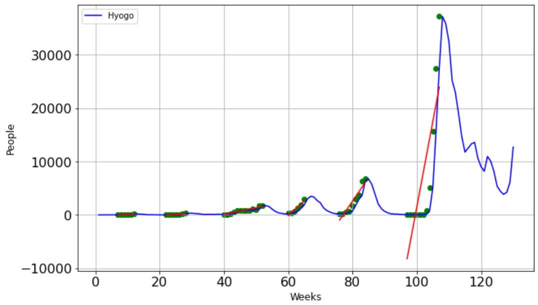

--- 29. Nara ---
1st [7.5] ,2nd [10.5] ,3th [19.] ,4th [102.4] ,5th [190.5] ,6th [1036.2]

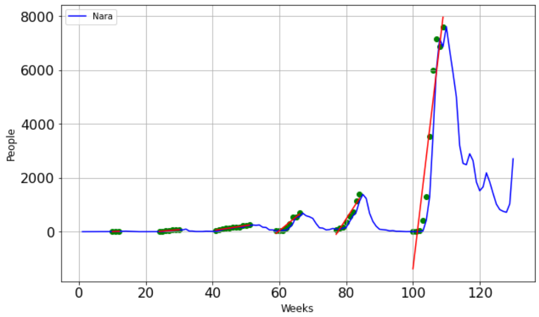

--- 30. Wakayama ---
1st [2.5] ,2nd [8.5] ,3th [5.5] ,4th [40.3] ,5th [72.8] ,6th [759.9]

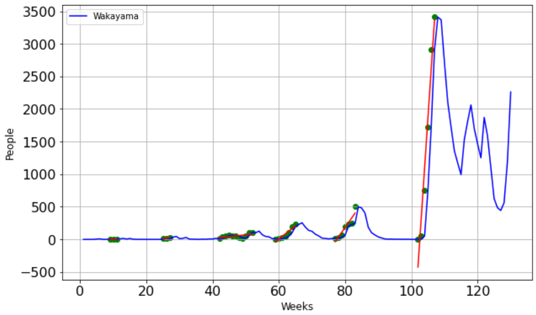

--- 31. Tottori ---
1st [1.] ,2nd [11.] ,3th [24.] ,4th [2.] ,5th [18.5] ,6th [121.1]

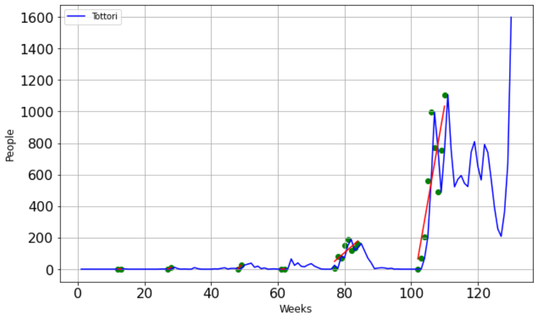

--- 32. Shimane ---
1st [8.] ,2nd [45.5] ,3th [3.6] ,4th [7.] ,5th [22.3] ,6th [192.1]

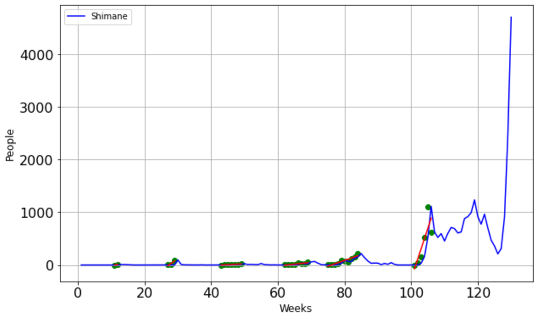

--- 33. Okayama ---
1st [6.] ,2nd [8.8] ,3th [36.2] ,4th [132.5] ,5th [220.1] ,6th [1592.]

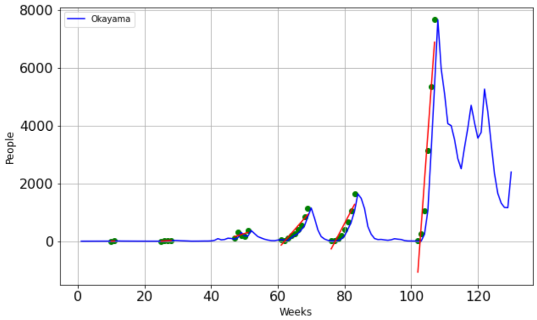

--- 34. Hiroshima ---
1st [14.1] ,2nd [23.2] ,3th [95.3] ,4th [147.8] ,5th [299.] ,6th [1881.4]

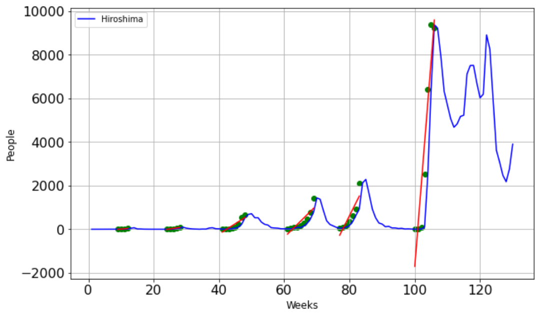

--- 35. Yamaguchi ---
1st [0.5] ,2nd [3.5] ,3th [24.5] ,4th [34.2] ,5th [109.2] ,6th [484.7]

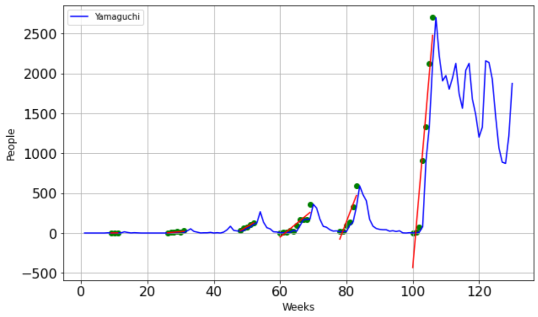

--- 36. Tokushima ---
1st [-0.2] ,2nd [10.4] ,3th [23.6] ,4th [47.] ,5th [47.8] ,6th [255.2]

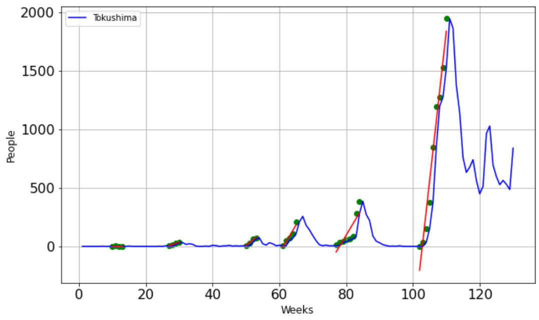

--- 37. Kagawa ---
1st [5.] ,2nd [14.] ,3th [27.9] ,4th [29.5] ,5th [99.1] ,6th [368.5]

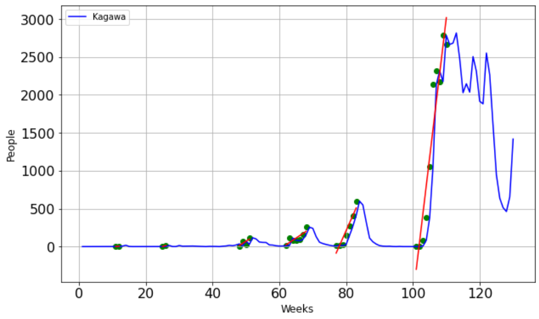

--- 38. Ehime ---
1st [16.] ,2nd [5.] ,3th [8.2] ,4th [42.4] ,5th [93.2] ,6th [560.5]

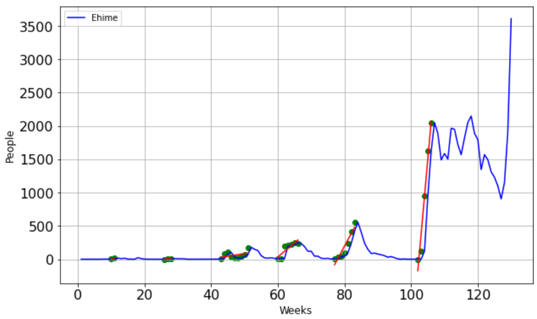

--- 39. Kochi ---
1st [17.] ,2nd [2.7] ,3th [44.5] ,4th [22.4] ,5th [50.2] ,6th [325.]

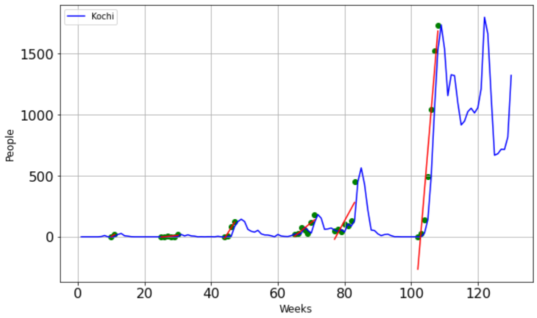

--- 40. Fukuoka ---
1st [71.] ,2nd [181.2] ,3th [164.4] ,4th [562.4] ,5th [1226.7] ,6th [4606.8]

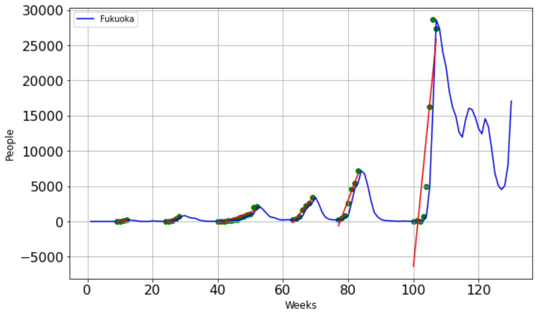

--- 41. Saga ---
1st [7.] ,2nd [18.5] ,3th [44.] ,4th [41.4] ,5th [163.8] ,6th [605.7]

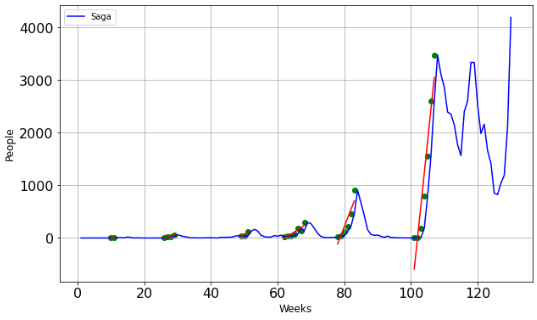

--- 42. Nagasaki ---
1st [8.] ,2nd [19.4] ,3th [60.] ,4th [73.1] ,5th [107.8] ,6th [689.9]

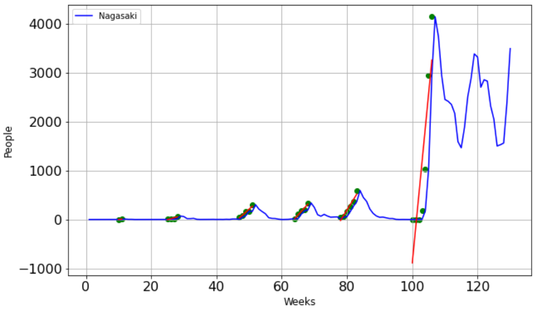

--- 43. Kumamoto ---
1st [1.5] ,2nd [77.5] ,3th [63.6] ,4th [96.5] ,5th [280.4] ,6th [1397.]

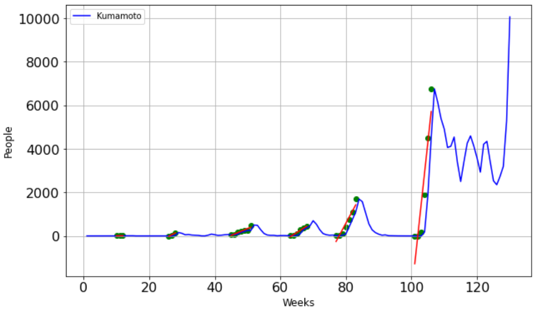

--- 44. Oita ---
1st [-8.] ,2nd [5.9] ,3th [9.8] ,4th [63.6] ,5th [139.9] ,6th [585.3]

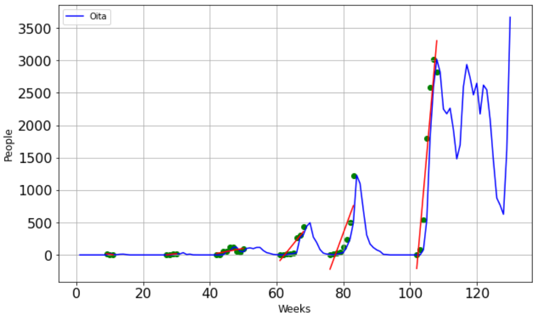

--- 45. Miyazaki ---
1st [1.5] ,2nd [26.9] ,3th [126.6] ,4th [57.7] ,5th [135.4] ,6th [689.4]

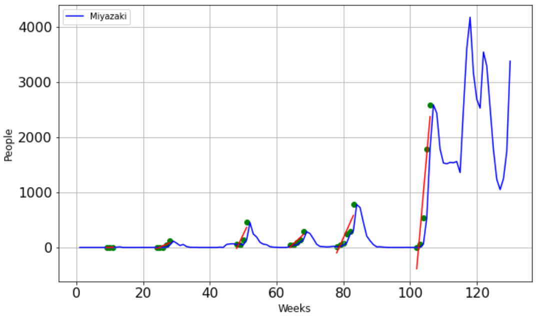

--- 46. Kagoshima ---
1st [0.8] ,2nd [87.] ,3th [20.2] ,4th [36.9] ,5th [282.2] ,6th [902.9]

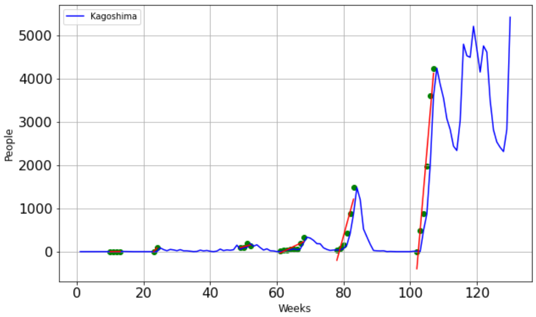

--- 47. Okinawa ---
1st [21.5] ,2nd [113.7] ,3th [99.7] ,4th [65.5] ,5th [896.9] ,6th [2575.1]

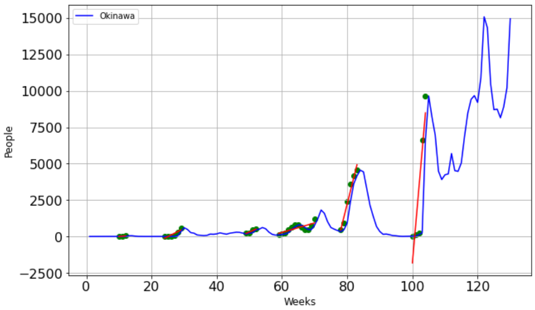

Supplement: Supplementary file 1 — Supplementary Material 1 [file 12879_2024_9354_MOESM1_ESM.pdf]
