# Supplementary material for: Simple mathematical model for predicting COVID-19 outbreaks in Japan based on epidemic waves with a cyclical trend
Source: BMC Infect Dis. 2024 May 9;24:465. doi: 10.1186/s12879-024-09354-5 (PMC11080248; doi:10.1186/s12879-024-09354-5)
Supplement: Supplementary file 2 — Supplementary Material 2 [file 12879_2024_9354_MOESM2_ESM.pdf]

Additional file 1.

S1. Table. Coefficients used in predicting the slope of the rising line

		1st wave	2nd wave	3rd wave	4th wave	5th wave	6th wave	7th wave
Epidemic Period	Rize	28	133	266	413	532	686	889
	Peak (b_i)	91	207	367	483	588	756	959
Slope of the rising line		345	787	2,366	3,879	21,538	69,103	229,523
Total number of infected persons (a_i)		16,494	62,843	352,881	351,203	937,559	4,300,000	14,000,000
Adjustment (c_i)		8	9	10	11	12	11	12
